# Operational drift: why validated fatigue induction is the missing link in nature restoration research

**DOI:** 10.3389/fpsyg.2026.1780136

**Published:** 2026-03-12

**Authors:** Brodie E. Mangan

**Affiliations:** Psychology, Faculty of Natural Sciences, University of Stirling, Stirling, United Kingdom

**Keywords:** attention restoration theory, cognitive fatigue, fatigue induction, methodological heterogeneity, nature exposure

## 1 Introduction

Despite robust evidence that contact with nature consistently elevates mood and wellbeing (McMahan and Estes, 2015), the cognitive record remains inconsistent. This paradox is illustrated by the largest systematic review of the field, which analyzed 80 experiments and reported only small average cognitive benefits, a 35% null result rate, and substantial heterogeneity (Bell et al., 2025).

This inconsistency is a recurring diagnosis. Ohly et al. (2016) questioned the empirical support underpinning Attention Restoration Theory (ART; Kaplan, 1995), highlighting substantial heterogeneity in study design, measures, and operational definitions of “attention,” alongside methodological limitations. They argued that variation in task demands and measurement sensitivity undermines confidence that many paradigms adequately operationalise directed attention fatigue. Stevenson et al. (2018) similarly highlighted “substantial ambiguity” in restoration operationalisation.

I contend these results reflect a methodological failure: the “operational drift” of cognitive fatigue induction. Kaplan (1995, p. 172) explicitly stated “restoration of effectiveness is at the mercy of recovery from directed attention fatigue”; yet the field drifted toward methods failing to verify this prerequisite. While reviews identified heterogeneity as a symptom, this paper identifies the mechanism: the shift from measuring performance to subjective state, and from inducing fatigue to assuming it. Without verification, the paradigm risks unfalsifiability.

A distinction between mental and cognitive fatigue clarifies the methodological requirements. Mental fatigue refers to subjective feelings of tiredness; cognitive fatigue refers to measurable performance impairment (Pessiglione et al., 2025). These states can dissociate: individuals may feel mentally fatigued while maintaining performance through compensation, or show impairment without subjective awareness. The construct of “directed attention” overlaps with what cognitive neuroscience terms executive functions—processes mediated by prefrontal-parietal networks (Holroyd, 2025; Pessiglione et al., 2025). The critical point is operational: if restoration claims concern cognitive processes, evidence must be cognitive. Subjective reports of feeling “refreshed” may reflect preference, expectation, or improved mood rather than restored function.

## 2 Operational drift: why “tired” is not enough

The fatigue-based paradigm is, in principle, already the standard: ART posits “directed attention fatigue” as central. However, empirical reality tells a different story. In their meta-analysis, Bell et al. (2025) provided quantitative evidence of this operational drift, finding that explicit fatigue induction was present in only 29% of studies.

Even among studies that claim to induce fatigue, many utilize tasks that are “fatiguing” in name only. Recent neuro-metabolic research explains why: sustained high-load cognitive work produces glutamate accumulation in the lateral prefrontal cortex, increasing the neural cost of cognitive control (Wiehler et al., 2022). This metabolic accumulation, not subjective tiredness, constitutes a physiological substrate of cognitive fatigue. Pessiglione et al. (2025) argue that brief, low-load tasks may never trigger sufficient metabolic change to impair prefrontal function; if no genuine deficit exists, there is nothing from which to restore. Influential studies have relied on Sustained Attention to Response Task (SART; a go/no-go paradigm measuring inhibitory control) durations as short as 5–10 min (e.g., Berto, 2005; Kimura et al., 2021). Emerging frameworks suggest these tasks induce subjective boredom (a motivational lapse) rather than cognitive fatigue (a functional constraint) (Holroyd, 2025; Mangin and Pageaux, 2025).

I examine Berto (2005) and Berman et al. (2008) not as isolated targets, but as archetypes that have failed replication attempts. Their protocols established conventions; brief induction tasks, absence of manipulation checks, reliance on post-exposure measurement, that subsequent studies widely replicated. Examining this evidential base is essential for understanding why convergence has proved elusive.

### 2.1 The Berto paradigm

Berto (2005) is cited as evidence that viewing natural images restores attentional function. Participants completed a 5-min SART, viewed images of nature, urban scenes, or geometric patterns, and repeated the SART. The nature group improved on *d*′ (a signal detection measure separating sensitivity from response bias; Macmillan and Creelman, 2005), interpreted as support for ART.

However, a 5-min vigilance bout is unlikely to induce metabolic fatigue, and no manipulation check was provided. Brief tasks induce boredom rather than functional impairment (Holroyd, 2025; Mangin and Pageaux, 2025).

The statistical pattern raises concerns. The nature group started lower than the urban group at baseline; by post-test, both converged to similar levels with no significant between-group difference on *d*′, more consistent with regression to the mean (the statistical tendency for extreme scores to move toward the average) than restoration. Neilson et al. (2021) found no restorative effect in direct replication. Without induced and verified fatigue, restoration cannot be distinguished from regression, rest, or relief from boredom.

### 2.2 The Berman paradigm

Much modern “restoration” literature traces to Berman et al. (2008), yet their paradigms do not establish a clean fatigue-restoration test. Without demonstrating impairment pre-exposure, post-exposure differences remain compatible with restoration, practice-based improvement, or shifts in arousal.

In Experiment 1, participants completed baseline measures and a 35-min directed forgetting task intended to “fatigue participants further” before walking in nature vs. an urban route. However, no objective performance assessment occurred after induction and before the walk. The key prerequisite, a verified pre-exposure decrement, is not demonstrated; post-walk improvement cannot distinguish recovery in nature from additional cognitive cost during the urban walk.

Experiment 2 used an underpowered within-subject design (*N* = 12): participants viewed nature or urban images for approximately 10 min between cognitive assessments using the Attention Network Task (ANT; a measure of alerting, orienting, and executive attention). The absence of validated fatigue induction and the short retest interval mean pre–post changes can reflect familiarization, regression, or effort allocation alongside any restoration process.

The authors concluded “directed-attention mechanisms were restored” (p. 1211), inferring successful fatigue induction. Yet without verified pre-intervention impairment, this interpretation remains uncertain. Johnson et al. (2021) conceptually replicated Experiment 2, finding no restorative effect on executive attention. Their accompanying meta-analysis (14 studies, *N* = 612) likewise found no reliable effect, suggesting the original study potentially conflated arousal or engagement with restoration.

### 2.3 Chronic issues

These issues persist. Marois et al. (2025) employed a rigorous design with subjective and objective measures, yet their 29-min fatigue induction produced no significant performance decline: reaction times unchanged, accuracy differences non-significant. Subjective fatigue increased, and following nature exposure participants reported feeling restored while cognitive performance showed no condition-specific benefit.

Xu et al. (2024) present a starker case: mental arithmetic induced fatigue under an ART framework, yet recovery was measured exclusively through heart rate variability, blood pressure, and self-report, with no cognitive outcome assessed. The cognitive component was entirely supplanted by stress physiology while retaining the language of attentional restoration.

These findings echo Kimura et al. (2021), where participants maintained above 90% accuracy while reporting increased subjective workload. Skin conductance decreased during nature video exposure, consistent with arousal reduction rather than cognitive recovery.

This dissociation is predictable given the mental–cognitive fatigue distinction. Subjective fatigue plausibly emerges earlier as a motivational signal than functional impairment (Holroyd, 2025; Mangan and Kourtis, 2026; Pessiglione et al., 2025). Brief, low-load tasks may elevate subjective strain without crossing into impaired cognition, precisely the zone where “restoration” claims become unfalsifiable.

### 2.4 Inadvertent fatigue and the enhancement question

Some studies report cognitive benefits following nature exposure without explicit fatigue induction, which might suggest that nature enhances cognition independently of restoration. However, closer examination often reveals inadvertent fatigue embedded in standard protocols. Bratman et al. (2015) administered 75 min of cognitive testing pre-walk; this extended battery likely induced substantial fatigue regardless of intention. Hartig et al. (2003) had participants drive 40 min to the field site, treating this as a low-demand baseline. Yet driving is a sustained cognitive task known to induce executive attentional withdrawal and objective performance errors (Benelli et al., 2024; Giorgi et al., 2024). Indeed, Hartig et al. (2003) acknowledged that the drive elevated participants' blood pressure significantly above baseline, an established marker of cognitive effort (Wright et al., 2008), consistent with inadvertent fatigue induction even in the non-fatigued condition.

Without manipulation checks, we cannot distinguish genuine cognitive enhancement from unrecognized fatigue-and-restoration. Restoration implies recovery from deficit; enhancement implies improvement beyond baseline. This distinction is important; as Joye and Dewitte (2018) noted, without a verified deficit, “restoration” becomes methodologically indistinguishable from simple vitality or motivation effects. Without a verified deficit, improvement should be classified as enhancement; ART's proposed mechanism cannot be conclusively evaluated.

## 3 Toward rigorous protocols

The trajectory is encouraging. Augustinova et al. (2022) isolated Stroop interference from facilitation and found Bayesian evidence against the claim that brief nature exposure reduces interference, despite participants rating images as restorative. Hartanto et al. (2023) employed a preregistered, high-powered within-subject design with open materials, yet found no benefit of nature videos on working memory. These outcomes are rigor doing its job by reducing the space of plausible explanations.

The remaining bottleneck is state definition. Participants may report feeling tired while performing normally (Holroyd, 2025; Kimura et al., 2021; Goodman et al., 2025). If participants are not objectively impaired before exposure, a null result is compatible with “no restoration required,” and a positive result with arousal or re-engagement rather than recovery.

Neurophysiological measures like EEG offer a path forward by directly indexing cognitive function. McDonnell and Strayer (2024) demonstrated changes in frontal midline theta (fmTheta) following nature immersion—a marker linked to cognitive control and fatigue (Mangan and Kourtis, 2026; Tran et al., 2020). However, interpretation requires care. The “theta paradox” illustrates that theta oscillations reflect near-opposite states (alert control and fatigue), making context critical (Snipes et al., 2022; Wascher et al., 2014). Consequently, post-nature fmTheta increases are compatible with restoration, fatigue, or compensation. Without behavioral anchoring, neural markers cannot discriminate between these interpretations.

The interpretive value of neurophysiological measures depends on first establishing behavioral impairment. If performance has not declined, neural changes cannot be attributed to cognitive recovery; they may reflect shifts in arousal, engagement, or affect. Behavioral impairment is the foundation; neurophysiology then clarifies mechanisms.

## 4 Discussion

Many reported cognitive “restoration” effects are simply not diagnostic of restoration. Restoration is a recovery claim, and recovery requires a verified pre-exposure decrement. Where fatigue is assumed rather than induced and measured, post-exposure improvements remain compatible with arousal-based enhancement, relief from boredom, expectancy effects, and individual differences in preference. These mechanisms can generate the same behavioral change as restoration, particularly in repeated-measures designs where practice gains and effort allocation are plausible.

Individual methodological recommendations have appeared in prior critiques (Ohly et al., 2016; Joye and Dewitte, 2018; Neilson et al., 2021). This paper integrates these within a falsifiability framework: without verified fatigue states, restoration claims become logically unfalsifiable. The field cannot adjudicate between ART and alternatives until studies demonstrate that (a) participants were genuinely fatigued, (b) nature exposure occurred, and (c) cognitive function was subsequently restored. All three are necessary; none are sufficient alone.

The practical solution is straightforward: combine a validated fatigue induction with an explicit manipulation check immediately before exposure, and include a neutral third control arm matched for movement and timing. This separates nature-related benefit from urban-related cost, and allows preference and expectancy to be measured rather than implicitly confounded.

At minimum, acute protocols should incorporate: (1) validated fatigue induction sufficient to produce a measurable decrement (demonstrated, not assumed); (2) manipulation check immediately before exposure confirming impairment; (3) appropriate controls (the common two-arm nature-vs.-urban design conflates potential benefit of nature with potential cost of urban exposure; a neutral third arm isolates the environmental variable); and (4) outcome measures sensitivity to cognitive fatigue, such as (*d'*) (Hopstaken et al., 2015), to distinguish genuine capacity loss from response bias. Where resources permit, neurophysiological indices may clarify mechanisms if anchored by this behavioral impairment.

## 5 Conclusion

Nature's cognitive benefits remain a legitimate scientific question, but the current evidence base cannot answer it cleanly. Decades of research produced small effects, substantial heterogeneity, and persistent replication difficulties; not because the hypothesis is wrong, but because methods have drifted from the conditions it requires. Until the field standardizes fatigue induction, manipulation checks, and appropriate controls, we will continue generating ambiguous data that cannot distinguish restoration from arousal, preference, or rest. The stakes extend beyond theory: claims about nature's cognitive benefits inform urban planning, healthcare design, and workplace interventions. These applications deserve an evidence base built on falsifiable tests.
